# Environmental Effects and Genetic Parameters for Growth Traits of Lohi Sheep

**DOI:** 10.3390/ani12243590

**Published:** 2022-12-19

**Authors:** Numan Sharif, Asad Ali, Muhammad Dawood, Muhammad Irfan-ur-Rehman Khan, Duy Ngoc Do

**Affiliations:** 1 Department of Animal Breeding and Genetics, University of Veterinary and Animal Sciences, Lahore 54000, Pakistan; 2 Department of Theriogenology, University of Veterinary and Animal Sciences, Lahore 54000, Pakistan; 3 Faculty of Veterinary Medicine, Viet Nam National University of Agriculture, Hanoi 100000, Vietnam; 4 Department of Animal Science and Aquaculture, Dalhousie University, Truro, NS B2N 5E3, Canada

**Keywords:** animal models, covariance, genetic correlation, heritability, Lohi sheep, permanent environment

## Abstract

**Simple Summary:**

Animal breeders are always interested in improving the economically important traits such as growth by selecting and breeding genetically superior animals. The Lohi sheep is one of Pakistan’s most important meat type breeds as it significantly contributes to the country’s total mutton production. To fulfil the higher demands of mutton production, there is a need to define optimum breeding strategies for improving growth in this breed. This study reported the moderate heritabilities for growth traits in Lohi sheep and significant influences of sex, year of birth and type of birth on these traits. The results might be useful for designing a breeding program for higher mutton production in Lohi sheep.

**Abstract:**

Estimating genetic parameters for growth traits is crucial to plan breeding strategies for improving meat production in indigenous sheep breeds. The study first tested the effects of environmental and maternal effects on five growth traits, including birth weight (BWT), weight at 120 days (WT120), weight at 180 days (WT180), weight at 270 days (WT270) and weight at 365 days of age (WT365) and then estimated genetic parameters for these traits using data obtained in 1215 Lohi sheep. The effects of factors, including year (YOB), season (SOB) and type of birth (TOB), age of dam (AOD) and sex on growth traits of Lohi sheep, were examined using analysis of variance (ANOVA) in R software. Sex, TOB and YOB significantly affected all studied traits. The estimates of direct and maternal heritability for BWT and WT120 were 0.15 ± 0.08 and 0.20 ± 0.06, and 0.45 ± 0.16, 0.21 ± 0.08, respectively. The direct heritability estimates for WT180, WT270 and WT365 were 0.20 ± 0.07, 0.21 ± 0.07 and 0.19 ± 0.08, respectively. Due to the high heritability estimate obtained for WT120 compared to other studied traits, and its strong genetic correlation (>0.9) with post-weaning growth traits, it is recommended that selection must be practiced on WT120 to improve the growth performance of Lohi sheep. The results could be used for the development of genetic/genomic selection programs aiming to improve the production performance of the Lohi sheep.

## 1. Introduction

The production of animals with higher growth rates is necessary to fulfil the increasing need for animal protein in the human diet. Growth of an animal is defined as the increase in size or volume of tissues over time [1,2]. The growth rate in farm animals can be improved through planned breeding based on genetic selection [3]. Estimated breeding values (EBVs) have been widely used to develop the selection index for selecting animals with high genetic merits. However, to accurately estimate EBVs, the appropriate (environmental and maternal) effects should be used in an animal model [4]. Growth is a complex trait which is known to be affected by genetic and environmental factors, as well as their interaction. Some factors that can influence early growth may include maternal environmental effects, the suckling period of lamb, milk production of the dam and feeding management of animals. All the influences of a dam on her offspring, except the influence of genes she transmitted to them, are known as maternal environmental effects [5]. These effects may arise due to the mothering ability of the dam and its milk production, cytoplasmic inheritance and the influence of the uterine environment [6]. The growth of lamb is also influenced by the genotype of a dam for maternal impacts and her additive genes for growth [7]. Some other environmental factors which affect growth traits in sheep include season of birth (SOB), type of birth (TOB), year of birth (YOB), age of dam (AOD) and sex of animals [8,9]. It is well documented that the maternal effect is crucial in estimating the direct heritability of growth and reproductive performance traits [10,11,12,13]. The animal models which do not account for maternal effects may overestimate direct heritability, leading to the biased prediction of response to selection [6,14,15,16].

The Lohi, a well-known thin-tailed sheep breed of Pakistan, is known for its mutton and wool production. It can be found throughout the Punjab province, but most of its population is in central Punjab. It contributes up to 40% of the total mutton production of the province [2,17]. It shows wide diversity in different production traits, so there are more chances for improvement in the performance of this breed [18]. In the past, Babar et al. [19] estimated heritability for birth weight (BWT) only, and Javed et al. [20] estimated heritability for BWT, weaning weight and yearling weight in Lohi sheep. However, the literature lacks information on maternal components of growth traits in Lohi sheep. Likewise, none of these studies estimated the genetic and phenotypic correlation of growth traits in the breed. The current study evaluated environmental maternal influences on five growth traits in Lohi sheep. It estimated the genetic and phenotypic parameters for these traits, which will be used to design an actual breeding program for the genetic selection of Lohi lambs.

## 2. Materials and Methods

### 2.1. Research Site and Flock Management

The study was executed at the Small Ruminant Training and Research Centre (SRT & RC) Pattoki, Punjab, Pakistan. Lohi sheep farming at SRT & RC was started in 2007 by purchasing 100 animals from Livestock Experiment Station, Bahadurnagar, Okara. The average age of Lohi lambs at weaning was four months. After weaning, males and females were separately raised in different sheds. Controlled breeding was practised on the farm. Males were exposed daily to females for 1 h during breeding seasons: Spring (February to April) and Autumn (September to November). The females that did not conceive during breeding seasons were again exposed to males. Animals were allowed to graze for 4 to 5 h daily on shrubs and seasonal forages. Seasonal forages were also provided in the chopped form to the animals after they returned to grazing. Additionally, concentrates were also provided to the pregnant, suckler and breeding males at the rate of 250 to 500 g/day, 100 to 150 g/day and 800 to 1000 g/day, respectively.

### 2.2. Statistical Analysis

The data regarding animal ID, birth type, sex, date of birth, pedigree and body weights on 1215 Lohi lambs born from 2008 to 2019 were collected from SRT & RC. The data were evaluated for the identification of outliers. The growth records which lied outside the mean ± 3 standard deviations were removed before the final analysis.

Because of variation in weaning ages, body weights were adjusted to 120 days of age. Similarly, due to age differences in days at 6, 9 and 12 months, body weights were corrected to 180, 270 and 365 days of age using the formula given below:

z=b+((w − b)/a) × d

z = adjusted weight for specific days of age (120, 180, 270 and 365),
b = birth weight,
w = weight recorded at 4, 6, 9 and 12 months,
a = age in days at the measurement of weight at 4, 6, 9 and 12 months,
d = 120, 180, 270 and 365 days accordingly for each trait.

The impact of different environmental sources of variation on growth traits, including birth weight (BWT), weaning weight (WT120), weight at 180 (WT180), weight at 270 (WT270) and weight at 365 days of age (WT365) were investigated through the analysis of variance (ANOVA) using the ‘car’ package [21] in R version 4.2.1 [22]. The studied environmental factors were the season of birth (SOB) with five levels (Spring: February to April; Hot Dry: May, June; Hot Humid: July, August; Autumn: September, October; Winter: November to January), year of birth (YOB) with twelve levels (2008 to 2019), sex with two levels (Male and Female), age of dam (AOD) with three levels (young: ≤2.5 years, mature: 2.5~4 years, old: >4 years) and type of birth (TOB) with two levels (single and twin). The factors found significant (*p* < 0.05) were used in models for the estimation of variance components. The least square (LS) means for each category within each factor were estimated using the lsmeans package of R [23]. Duncan’s multiple range test was performed to analyse the variation among different groups in each factor using duncan.test function of the agricolae package in R [24].

### 2.3. Estimation of Variance Components

We used six animal models accounting for or ignoring maternal permanent environmental effect, maternal genetic effect or their combination in the univariate analysis, performed through the restricted maximum likelihood method (REML) of WOMBAT to estimate covariance components and heritability of growth traits [25]. 

Model 1

$$Y=Xb+Z_{a}\;a+e$$

Model 2

(1) $$Y=Xb+Z_{a}\;a+Z_{m}\;m+e\;\mathrm{with}\;\mathrm{Cov}\left(Z_{m},\;m_{o}\right)=0$$

Model 3

(2) $$Y=Xb+Z_{a}\;a+Z_{m}\;m+e\;\mathrm{with}\;\mathrm{Cov}\left(Z_{m},\;m_{o}\right)=A\sigma_{m}$$

Model 4

(3) $$Y=Xb+Z_{a}\;a+Z_{c}\;c+e\;$$

Model 5

(4) $$Y=Xb+Z_{a}\;a+Z_{m}\;m+Z_{c}\;c+e\;\mathrm{with}\;\mathrm{Cov}\left(Z_{m},\;m_{o}\right)=0$$

Model 6

$$Y=Xb+Z_{a}\;a+Z_{m}\;m+Z_{c}\;c+e\;\mathrm{with}\;\mathrm{Cov}\left(Z_{m},\;m_{o}\right)=A\sigma_{m}$$

where Y is the vector of observation for each growth trait, b is the vector of unknown fixed effects with incidence matrix X, a is the vector for additive direct genetic effect with incidence matrix Z_a_, m is the vector of maternal additive genetic effect and Z_m_ is the incidence matrix linked to it, c is the vector of maternal permanent environmental effect with incidence matrix Z_c_, e is the vector for residual effects, A is the numerator relationship matrix between animals and $\sigma_{\mathrm{am}}$ is the covariance between additive direct and maternal genetic effects. The variance (V) and covariance (COV) structure for matrices involving random effects was assumed as:

$${V(a)=A\sigma }^{2}a,\;{V(m)=A\sigma }^{2}m,\;{V(c)=I\sigma }^{2}c,\;V\left(e\right){=I\sigma }^{2}e\;\mathrm{and}\;{\mathrm{COV}(a,m)=A\sigma }_{\mathrm{am}}$$

where I represents the identity matrix, $\sigma^{2}a$ is the additive direct variance, $\sigma^{2}m$ additive maternal genetic variance, $\sigma^{2}c$ is maternal permanent environmental variance and ${I\sigma }^{2}e$ is residual variance. The direct maternal correlation $(r_{\mathrm{am}}$) was obtained as $\sigma_{\mathrm{am}}/\left(\sigma_{a}\;\times \;\sigma_{m}\right)$.

The log-likelihood ratio test was applied to find an appropriate animal model for each growth trait. The Chi-square test (X^2^) was utilised to announce the significant variation between the two models and was calculated using the formula given below as described by [26]:

$$X^{2}{=-2(\mathrm{LogL}}_{r}{\;-\;\mathrm{LogL}}_{f})$$

where ${\mathrm{LogL}}_{r}$ and ${\mathrm{LogL}}_{f}$ represent the log-likelihood for basic and complete models, respectively, and $X^{2}$ is distributed as a combination of two $X^{2}$ distributions: $0{.5X}_{\mathrm{df},\;\alpha }^{2}$ + $0{.5X}_{0,\alpha }^{2}$, where df is the degrees of freedom computed as the difference in the number of parameters (random effects) in two models and α is alpha set at (0.05). A model with the least number of parameters was selected when the Chi-square resulted in non-significant differences.

Additionally, bivariate analyses using model 1 were done in WOMBAT to estimate the genetic and phenotypic correlations between growth traits.

## 3. Results and Discussion

### 3.1. Analysis of Environmental Effects of Growth Traits

The descriptive statistics of growth traits of Lohi sheep are shown in Table 1. The LS means for each category of Lohi animals are presented in Table 2 and Table 3. The significant (*p* < 0.001) variation in the body weights of lambs born in different years could be explained due to differences in environmental conditions, management of the farm and the availability of forages throughout these years. Factors like climatic conditions, availability of nutrients, fodder quality and disease prevalence can affect an animal’s growth performance. The maximum performance of an animal can only be attained if it is provided with good-quality nutrients, comfortable surroundings and disease control. As farm resources did not remain constant during the study period (12-year span), the availability of resources could have influenced the decisions of the farm manager, and, as a result, animal management was affected. Similarly, the availability of grasses to the lambs for grazing also depends on various factors, including rainfall. Hence, collectively all of these factors could have contributed to the significant variation in lambs’ growth performance in different years of study. The significant variation in growth traits of lambs born during different years observed in the current study was similar to previous studies on sheep [9,27,28,29].

The studied growth traits, except BWT and WT365, were significantly affected by SOB (Table 2). The insignificant influence of SOB on BWT was also observed in Kajli, Thalli and Dorper crossbred sheep [30,31,32]. Likewise, the non-significant influence of SOB on WT365 agreed with the findings of Zaffer et al. [31] in Dorper crossbred sheep. Contrary to the current study, Momoh et al. [33] and Mohammadi and Latifi [34] reported a significant effect of SOB on WT365 in different sheep breeds. The LS means of lambs born in autumn were slightly higher (3.09 kg) but not significantly different to those born in spring (3.05 kg). The difference in the growth performance of animals born in different seasons might be due to the difference in the availability of nutrients during these seasons. The animals were mainly fed with green forages, and their availability was inconsistent in different seasons of the year. The forages like berseem (*Trifolium alexandrinum*) and oats (*Avena sativa*) were excessively available during the winter and spring seasons at the farm. Hence, dams which completed their gestation during these seasons had a better chance to avail more fresh fodder, which had an effect on their milk performance and lambs belonging to these dams were found heavier at weaning. The significant influence of SOB on some growth traits agreed with previous findings [31,33,34,35].

Lamb’s sex significantly influenced all the studied traits (Table 3). Male lambs had higher LS means compared to females for all the traits. The male lambs were also found heavier at various age points in studies published by Bahreini Behzadi et al. [36], Rahimi et al. [9] and Tohidi et al. [28] on Kermani, Makuie and Iran-Black sheep, respectively. A possible reason for males being heavier at birth is explained by Benyi et al. [37], who stated that male lambs grow faster in the uterus than females. Similarly, Babar et al. [38] reported that pregnancy duration for male lambs is slightly longer than for females. The other possible reason for variation in the body weights of males and females might be hormonal differences. The influence of sex hormones becomes more prominent as the animal reaches maturity. In males, testosterone is produced in larger quantities, acting as a growth enhancer [39]. Whereas in females, the significant hormones are oestrogen and progesterone. Oestrogen has a restricted influence on the growth of long bones [33].

Dam’s age only significantly affected weights at early ages, i.e., BWT, WT120 and WT180 (Table 3). Some other studies also reported a significant influence of AOD on pre-weaning growth traits, which is in line with present findings [9,32,34,40,41]. The non-significant effect of AOD on post-weaning traits observed herein was also in line with previous studies [9,33,42,43,44]. Contrary to this, Rahimi et al. [9], Naderi [41] and Bahreini Behzadi et al. [36] observed a significant effect of AOD on WT180 and WT270 in Makuie and Kermani sheep, respectively. In the present study, we observed that younger ewes produced lighter lambs, indicating their physiological events. As younger dams and their organs are still under development, they might not support larger foetuses. Their energy was not only utilised in the development of foetuses present in their uteruses, but also in their own development [38]. As mature and older ewes were thoroughly developed, most of their energy was utilised in foetal development, producing heavier lambs at birth, contrary to younger ewes. The ewes which become dams at younger ages produce less milk because their udder is not completely developed at that time. So, less milk is available to the lambs born from these dams compared to those born from aged ewes, and the impact of this fact was clearly observed in the weaning weight of lambs.

We observed a significant influence of TOB on BWT, WT120, WT180, WT270 and WT365 (Table 3). The single-born lambs were found to be heavier than twins for these traits. Some previous studies also reported single-born as heavier than twins [33,35,37,45]. One reason for singles being heavier at birth can be explained by the fact of availability of space and nutrients in the uterus [38]. The single-born lambs do not compete for space and nutrients in the dam’s womb. Therefore, their growth was faster in the uterus, and they attained higher BWT than twins. In the case of twins, they had to struggle to get nutrients and space in the uterus, and due to less accessibility of both nutrients and space, they were lighter at birth than single-born lambs. After birth, twins again had to face competition to access milk from the dam, and as a result, their weights were again lower at weaning. On the other hand, each lamb gets an equal chance to take nutrients from the feedlot during the post-weaning stages. Even though the body weights of twins were lower than that of singles for post-weaning growth traits, which might be due to their compromised growth at early ages. 

### 3.2. Estimation of Variance Components and Heritability

The log-likelihood test revealed Model 3 as the best equation for BWT and WT120 (Table 4). This model represents direct and maternal additive genetic effects, including covariance between them. In agreement with previous findings, the permanent maternal environment did not significantly affect the preweaning growth traits [46,47]. The animal model only including direct additive genetic effect was the best equation for WT180, WT270, and WT365. The estimates of variance components and corresponding heritability obtained by six different equations for all studied growth traits of Lohi sheep are represented in Table 5.

The estimate of direct heritability attained by model 3 for BWT was 0.15 ± 0.08. These results were close to values of 0.10 and 0.11 reported by Babar et al. [19] and Javed et al. [20] in Lohi sheep, respectively. A similar estimated heritability (0.14) was also reported by Qureshi et al. [32] in Kajli sheep. However, relatively higher estimates of heritability for BWT were also reported by other studies, such as values of 0.28 for Ghezel sheep [11], 0.32 for Harnai sheep [48], 0.39 for Mengali sheep [35] and 0.39 for Djallonke sheep [49]. Meanwhile, lower heritabilities for BWT were reported by Balasubramanyam et al. [50] and Boujenane and Diallo [51] for Madras Red (0.08) and Sardi sheep (0.07), respectively. The estimates of direct heritability for BWT in Pelibuey and Blackbelly sheep were very low (0.01 and 0.05, respectively) [52]. The estimate of maternal heritability for BWT herein was moderate and agreed with previous findings [47,53]. 

The direct and maternal heritability estimates for WT120 were high and moderate, respectively. The high heritability for WT120 indicates that selection for heavy-weight animals at weaning will improve the growth performance of Lohi sheep. Similar to the present study, higher direct heritability estimates were observed in Madras Red, Menz and Djallonke sheep [49,50,54]. Contrary to this, several workers reported lower estimates ranging from 0.03 to 0.17 in different sheep breeds [4,14,32,55,56]. The literature found lower to moderate maternal heritability for weaning weight [14,57,58]. The moderate maternal heritability estimate of weaning weight indicates that this trait is not only dependent on the lamb’s own genetic potential, but also on the mothering ability and milk production of the dam. Since measuring ewes’ milk production is not a common practice in most sheep enterprises in Pakistan, the weaning weight of lambs could be the selection criteria [59]. MacNeil et al. [60] reported that selection based on EBVs for maternal pre-weaning gain could be as effective in improving milk yield as direct selection. 

The magnitude of the relationship between direct and maternal genetic effects for BWT and WT120 was highly negative, indicating the significant role of maternal genetic effect in the pre-weaning growth traits of Lohi sheep. It also suggests that improving one effect consequently decreases the other one. According to Szabó et al. [59], by selecting sires only on their direct EBVs without taking into account maternal EBVs, the weaning weights of their grand offspring will decrease, and therefore, no genetic progress will be expected from this selection. Moreover, adding negative additive-maternal covariance in the equation improved these traits’ direct and maternal heritability. A negative correlation between direct and maternal genetic components was also found in Nellore [56] and Muzaffarnagari sheep [61].

Post-weaning growth traits of Lohi sheep were not significantly affected by maternal genetic and permanent environmental effects, limiting maternal effects to pre-weaning stages. The direct heritability for WT180 was estimated as 0.20 ± 0.07, similar to the findings of Kumar et al. [62] in Deccani Sheep and Akhtar et al. [14] in Buchi sheep. However, very low estimates were also reported by Hussain et al. [63] in Thalli (0.07), Mandal et al. [61] in Muzaffarnagari (0.06), Naderi [64] in Kurdi sheep (0.06) and Senemari et al. [65] in Zandi sheep (0.047). Some other authors found comparatively higher estimates ranging between 0.24 to 0.51 for the trait [42,50,53,54,66,67,68,69,70].

The heritability estimate (0.21 ± 0.07) for WT270 in the current study was in the range of earlier reports. The estimates for Makouei, Malpura, Muzaffarnagari, Zandi and Nellore sheep ranged between 0.1 to 0.16 [34,41,53,61]. On the other hand, relatively higher estimates were also observed in different breeds of sheep, such as heritabilities of 0.25, 0.27, 0.28, 0.30, 0.37, 0.45 and 0.49 were reported for Zandi, Nilagiri, Deccani, Madras Red, Mehraban, Ghezel and Santa Ines sheep breeds, respectively [11,34,42,50,62,66,68].

The estimate of heritability for WT365 was 0.19 ± 0.08, which was in the range from 0.14 to 0.43 reported in other sheep breeds, including Muzaffarnagari, Ghezel, Djallonke, Pak-Awassi, Nilagiri, Harnali, Deccani, Horro and Harnai [11,48,49,55,61,62,66,69,71]. Overall, the growth traits of Lohi sheep had low to moderate heritability. These results indicate that these traits are under some genetic control and can be used for selection purposes. Moreover, the higher heritability estimated for WT120 shows that selection for this trait could improve growth and mothering ability in Lohi sheep.

### 3.3. Genetic and Phenotypic Correlations

Several bivariate analyses were performed to estimate the genetic and phenotypic correlations between the growth traits of Lohi sheep. The results of the analyses are represented in Table 6. The genetic correlation estimates between most of the growth traits of Lohi sheep were strongly positive. Unexpectedly, BWT was found to be negatively genetically correlated with WT270, indicating that the selection on BWT may decrease WT270. Magotra et al. [72] stated that a negative genetic correlation of BWT with post-weaning growth traits might arise due to a significant decrease in maternal genetic effects and partly due to increased additive genetic variance. A positive phenotypic correlation was observed among all the studied traits. The phenotypic correlation between traits arises because of their shared common environment [29]. The highest genetic correlation (>0.9) of weaning weight with all the post-weaning traits suggests that these traits are either controlled by the same set of genes or the genes controlling these traits are strongly linked. Hence, the selection on WT120 may significantly improve the post-weaning growth traits in Lohi sheep. Most of the earlier studies also found positive genetic and phenotypic correlations between growth traits in sheep [8,12,53,58,73].

## 4. Conclusions

The results suggested that environmental factors like SOB, YOB, AOD, TOB and sex were significant sources of differences in the growth performance of Lohi sheep. So, it is recommended that known environmental factors must be considered while defining a model to estimate genetic parameters. The maternal effect only influenced the pre-weaning growth traits and did not influence post-weaning growth traits, suggesting that the effect of maternal components should be considered to estimate unbiased genetic parameters for pre-weaning growth traits. The highest heritability estimate obtained for weaning weight and strong genetic correlation of weaning weight with post-weaning traits suggests that selection could be fruitful if it is based on the weaning weight of Lohi sheep.

## Figures and Tables

**Table 1:** Descriptive statistics of growth traits of Lohi sheep.

Trait	No. of Animals	No. of Sires	No. of Dams	Mean (Kg)	SD
BWT	1215	40	380	3.35	0.86
WT120	1076	40	356	16.25	4.95
WT180	1010	39	348	20.26	5.66
WT270	894	37	328	24.62	6.15
WT365	803	37	311	28.73	6.89

BWT: birth weight; WT120: weight at 120 days of age; WT180: weight at 180 days of age; WT270: weight at 270 days of age; WT365: weight at 365 days of age; SD: standard deviation.

**Table 2:** Effect of season and year of birth on growth traits of Lohi sheep.

Factors	BWT (kg)		WT120 (kg)		WT180 (kg)		WT270 (kg)		WT365 (kg)	
	N	LSM ± SE	N	LSM ± SE	N	LSM ± SE	N	LSM ± SE	N	LSM ± SE
Season of Birth	NS		***		***		***		NS	
Spring	640	3.05 ^a^ ± 0.03	569	15.39 ^a^ ± 0.23	528	19.40 ^a^ ± 0.18	458	23.17 ^b^ ± 0.30	418	28.26 ^ab^ ± 0.36
Hot Dry	41	2.99 ^b^ ± 0.11	36	14.01 ^b^ ± 0.71	33	16.53 ^b^ ± 0.52	32	22.16 ^b^ ± 0.93	29	28.03 ^ab^ ± 1.07
Hot Humid	119	2.98 ^b^ ± 0.06	96	13.19 ^b^ ± 0.44	84	16.73 ^b^ ± 0.34	79	22.23 ^b^ ± 0.60	70	27.90 ^b^ ± 0.69
Autumn	146	3.09 ^a^ ± 0.06	125	14.68 ^a^ ± 0.41	119	18.90 ^a^ ± 0.30	115	24.81 ^a^ ± 0.52	99	29.23 ^a^ ± 0.61
Winter	165	2.91 ^b^ ± 0.05	149	15.89 ^a^ ± 0.36	142	20.22 ^a^ ± 0.28	137	25.21 ^a^ ± 0.46	120	28.03 ^a^ ± 0.55
Year of Birth	***		***		***		***		***	
2008	27	2.77 ^fg^ ± 0.13	21	12.76 ^e^ ± 0.91	17	16.91 ^cde^ ± 0.71	18	21.48 ^cd^ ± 1.23	15	25.21 ^d^ ± 1.47
2009	92	2.69 ^fg^ ± 0.07	76	13.24 ^e^ ± 0.48	62	17.26 ^cde^ ± 0.39	53	22.30 ^c^ ± 0.71	40	27.01 ^c^ ± 0.90
2010	78	2.41 ^g^ ± 0.08	57	12.65 ^e^ ± 0.57	52	15.35 ^e^ ± 0.45	43	22.50 ^d^ ± 0.82	40	26.11 ^cd^ ± 0.93
2011	102	3.11 ^bcde^ ± 0.07	92	17.32 ^ab^ ±0.46	87	21.66 ^a^ ± 0.34	68	26.68 ^b^ ± 0.66	53	33.62 ^a^ ± 0.80
2012	107	3.58 ^a^ ± 0.06	94	18.87 ^a^ ± 0.42	88	23.11 ^a^ ± 0.31	85	26.94 ^b^ ± 0.56	83	31.07 ^b^ ± 0.62
2013	135	3.35 ^ab^ ± 0.06	114	15.50 ^cd^ ± 0.41	107	18.23 ^bc^ ± 0.30	103	20.93 ^cd^ ± 0.54	97	23.74 ^d^ ± 0.62
2014	141	3.14 ^bcd^ ± 0.06	120	12.84 ^e^ ± 0.44	99	15.37 ^de^ ± 0.35	87	20.57 ^cd^ ± 0.62	80	27.58 ^c^ ± 0.72
2015	89	2.88 ^ef^ ± 0.07	81	14.08 ^de^ ± 0.48	81	17.48 ^bcd^ ± 0.35	79	22.86 ^c^ ± 0.62	76	25.56 ^cd^ ± 0.69
2016	118	3.27 ^bc^ ± 0.07	112	13.27 ^e^ ± 0.43	106	16.20 ^cde^ ± 0.32	104	21.24 ^cd^ ± 0.56	79	24.16 ^d^ ± 0.68
2017	74	3.11 ^bcd^ ± 0.08	72	15.30 ^bc^ ± 0.52	72	18.28 ^b^ ± 0.37	68	27.49 ^ab^ ± 0.68	68	33.98 ^a^ ± 0.76
2018	123	3.00 ^cde^ ± 0.06	119	15.30 ^bc^ ± 0.42	118	29.97 ^a^ ± 0.31	113	27.69 ^a^ ± 0.55	105	33.13 ^a^ ± 0.64
2019	25	2.75 ^de^ ± 0.13	17	14.12 ^cd^ ± 1.00	17	20.40 ^a^ ± 0.72				

BWT: birth weight; WT120: weight at 120 days of age; WT180: weight at 180 days of age; WT270: weight at 270 days of age; WT365: weight at 365 days of age; LSM: least squared mean; SE: standard error; Spring: February to April; Hot Dry: May, June; Hot Humid: July, August; Autumn: September, October; Winter: November to January; N: number of animals; NS: Non-significant (*p* > 0.05); *** (*p* < 0.001); different superscripts within a column under a factor represents significant differences among different levels (*p* < 0.05).

**Table 3:** Effect of sex, age of dam and type of birth on growth traits of Lohi sheep.

Factors	BWT (kg)		WT120 (kg)		WT180 (kg)		WT270 (kg)		WT365 (kg)	
	N	LSM ± SE	N	LSM ± SE	N	LSM ± SE	N	LSM ± SE	N	LSM ± SE
Sex	***		***		***		***		***	
Female	570	2.93 ^b^ ± 0.03	512	14.12 ^b^ ± 0.27	479	17.79 ^b^ ± 0.22	462	22.66 ^b^ ± 0.35	422	27.05 ^b^ ± 0.40
Male	541	3.08 ^a^ ± 0.04	463	15.15 ^a^ ± 0.29	427	18.92 ^a^ ± 0.21	359	24.37 ^a^ ± 0.36	314	29.54 ^a^ ± 0.43
Age of Dam	***		***		***		NS		NS	
Young	353	2.84 ^b^ ± 0.05	310	13.81 ^b^ ± 0.31	283	17.54 ^b^ ± 0.25	255	22.91 ^b^ ± 0.40	227	27.05 ^b^ ± 0.47
Mature	400	3.09 ^a^ ± 0.04	344	15.26 ^a^ ± 0.30	322	18.68 ^a^ ± 0.23	293	23.83 ^a^ ± 0.40	258	28.37 ^b^ ± 0.46
Old	358	3.08 ^a^ ± 0.04	321	14.82 ^a^ ± 0.31	301	18.84 ^a^ ± 0.24	273	23.80 ^a^ ± 0.40	251	28.81 ^a^ ± 0.46
Type of Birth	***		***		***		***		***	
Single	892	3.44 ^a^ ± 0.03	794	16.18 ^a^ ± 0.22	744	19.98 ^a^ ± 0.16	671	25.04 ^a^ ± 0.27	595	29.28 ^a^ ± 0.32
Twin	219	2.57 ^b^ ± 0.05	181	13.08 ^b^ ± 0.36	162	16.72 ^b^ ± 0.30	150	21.98 ^b^ ± 0.46	141	27.30 ^b^ ± 0.53

BWT: birth weight; WT120: weight at 120 days of age; WT180: weight at 180 days of age; WT270: weight at 270 days of age; WT365: weight at 365 days of age; LSM: least squared mean; SE: standard error; Young: ≤2.5 years; Mature: 2.5~4 years; Old: >4 years; NS: Non-significant (*p* > 0.05); *** (*p* < 0.001); different superscripts within a column under a factor represents significant differences among different levels (*p* < 0.05).

**Table 4:** Model comparison based on log-likelihood ratios test for growth traits of Lohi sheep.

Trait	Model	Log L	Compared	LRT	DF	*p*-Value
BWT	Model 1	−91.73	1 vs. 6	17.74	3	<0.001
	Model 2	−88.00	2 vs. 6	10.28	2	<0.01
	Model 3	−83.13	3 vs. 6	0.54	1	0.462
	Model 4	−87.85	4 vs. 6	9.98	2	<0.01
	Model 5	−87.38	5 vs. 6	9.04	1	<0.01
	Model 6	−82.86				
WT120	Model 1	−1817.95	1 vs. 6	10.50	3	<0.01
	Model 2	−1817.54	2 vs. 6	9.68	2	<0.01
	Model 3	−1812.71	3 vs. 6	0.02	1	0.89
	Model 4	−1817.67	4 vs. 6	9.94	2	<0.01
	Model 5	−1817.53	5 vs. 6	9.66	1	<0.001
	Model 6	−1812.70				
WT180	Model 1	−1766.27	1 vs. 6	5.60	3	0.13
	Model 2	−1765.99	2 vs. 6	5.04	2	0.08
	Model 3	−1764.32	3 vs. 6	1.70	1	0.19
	Model 4	−1765.38	4 vs. 6	3.82	2	0.15
	Model 5	−1765.38	5 vs. 6	3.82	1	0.05
	Model 6	−1763.47				
WT270	Model 1	−1711.48	1 vs. 6	5.76	3	0.12
	Model 2	−1711.46	2 vs. 6	5.72	2	0.06
	Model 3	−1710.01	3 vs. 6	2.82	1	0.09
	Model 4	−1710.59	4 vs. 6	3.98	2	0.14
	Model 5	−1710.59	5 vs. 6	3.98	1	0.05
	Model 6	−1708.60				
WT365	Model 1	−1602.45	1 vs. 6	0.72	3	0.87
	Model 2	−1602.22	2 vs. 6	0.26	2	0.88
	Model 3	−1602.22	3 vs. 6	0.26	1	0.61
	Model 4	−1602.12	4 vs. 6	0.06	2	0.97
	Model 5	−1602.09	5 vs. 6	0	1	1.00
	Model 6	−1602.09				

BWT: birth weight; WT120: weight at 120 days of age; WT180: weight at 180 days of age; WT270: weight at 270 days of age; WT365: weight at 365 days of age; Log L: log-likelihood; LRT: $X^{2}$ test statistic for likelihood ratio test; DF: degrees of freedom for $X^{2}$ test.

**Table 5:** Estimates of variance components and related parameters obtained from six different models for growth traits of Lohi sheep.

Trait	Model	V_a_	V_c_	V_m_	V_e_	V_p_	σ_am_	H^2^ ± SE	C^2^ ± SE	M^2^ ± SE	r_am_
BWT	1	0.04			0.36	0.41		0.10 ± 0.05			
	2	0.02		0.03	0.36	0.41		0.05 ± 0.04		0.07 ± 0.03	
	3	0.06		0.08	0.34	0.41	−0.06	0.15 ± 0.08		0.20 ± 0.06	−0.95
	4	0.02	0.03		0.36	0.41		0.05 ± 0.04	0.07 ± 0.03		
	5	0.02	0.02	0.01	0.36	0.41		0.05 ± 0.04	0.05 ± 0.04	0.02 ± 0.04	
	6	0.06	0.01	0.07	0.34	0.41	−0.06	0.14 ± 0.08	0.03 ± 0.04	0.17 ± 0.08	−0.98
WT120	1	3.08			12.67	15.75		0.20 ± 0.07			
	2	2.66		0.45	12.61	15.72		0.17 ± 0.07		0.03 ± 0.03	
	3	7.36		3.51	9.86	16.35	−4.38	0.45 ± 0.16		0.21 ± 0.08	−0.87
	4	2.74	0.41		12.54	15.69		0.18 ± 0.07	0.03 ± 0.03		
	5	2.65	0.08	0.39	12.59	15.72		0.17 ± 0.07	0.01 ± 0.05	0.03 ± 0.04	
	6	7.37	0.15	3.36	9.82	16.34	−4.36	0.45 ± 0.16	0.01 ± 0.05	0.21 ± 0.10	−0.88
WT180	1	3.84			14.95	18.79		0.20 ± 0.07			
	2	3.36		0.46	14.93	18.75		0.18 ± 0.08		0.02 ± 0.04	
	3	6.50		2.47	13.07	19.14	−2.90	0.34 ± 0.14		0.13 ± 0.08	−0.73
	4	3.22	0.97		14.52	18.71		0.17 ± 0.07	0.05 ± 0.04		
	5	3.22	0.97	0.00	14.52	18.71		0.17 ± 0.07	0.05 ± 0.05	0.00 ± 0.04	
	6	6.79	1.39	1.39	12.38	19.16	−2.79	0.35 ± 0.15	0.07 ± 0.05	0.07 ± 0.07	−0.91
WT270	1	5.28			19.57	24.86		0.21 ± 0.07			
	2	5.07		0.18	19.58	24.83		0.20 ± 0.09		0.01 ± 0.04	
	3	8.86		2.56	17.35	25.26	−3.51	0.35 ± 0.15		0.10 ± 0.08	−0.74
	4	4.25	1.32		19.11	24.68		0.17 ± 0.08	0.05 ± 0.04		
	5	4.25	1.32	0.00	19.11	24.69		0.17 ± 0.08	0.05 ± 0.05	0.00 ± 0.05	
	6	8.92	1.95	1.23	16.44	25.23	−3.32	0.35 ± 0.15	0.07 ± 0.05	0.05 ± 0.08	−1.00
WT365	1	5.74			24.12	29.86		0.19 ± 0.08			
	2	4.36		0.90	24.39	29.67		0.15 ± 0.09		0.03 ± 0.04	
	3	4.36		0.92	24.39	29.66	−0.01	0.15 ± 0.11		0.03 ± 0.06	−0.01
	4	4.73	1.14		23.81	29.68		0.16 ± 0.08	0.04 ± 0.05		
	5	4.41	0.91	0.35	23.98	29.64		0.15 ± 0.09	0.03 ± 0.06	0.01 ± 0.05	
	6	4.19	0.92	0.22	24.10	29.62	0.17	0.14 ± 0.11	0.03 ± 0.06	0.01 ± 0.07	0.18

BWT: birth weight; WT120: weight at 120 days of age; WT180: weight at 180 days of age; WT270: weight at 270 days of age; WT365: weight at 365 days of age; V_a_: additive direct variance; V_c_ permanent maternal environment variance; V_m_: additive maternal variance; V_e_: residual variance; V_p_: phenotypic variance; σ_am_: covariance between additive direct and maternal genetic effects; c^2^: maternal permanent environment heritability; m^2^: maternal heritability; r_am_: correlation between direct and maternal additive genetic effects.

**Table 6:** Estimates of the genetic and phenotypic correlations between growth traits of Lohi sheep.

Trait 1	Trait 2	Genetic Correlation	Phenotypic Correlation
BWT	WT120	0.66 ± 0.06	0.59 ± 0.01
BWT	WT180	0.49 ± 0.10	0.36 ± 0.05
BWT	WT270	−0.45 ± 0.62	0.12 ± 0.04
BWT	WT365	0.35 ± 0.83	0.18 ± 0.04
WT120	WT180	0.93 ± 0.06	0.84 ± 0.01
WT120	WT270	0.91 ± 0.09	0.70 ± 0.02
WT120	WT365	0.94 ± 0.11	0.62 ± 0.02
WT180	WT270	0.92 ± 0.06	0.81 ± 0.01
WT180	WT365	0.94 ± 0.08	0.71 ± 0.02
WT270	WT365	0.86 ± 0.10	0.78 ± 0.02

BWT: birth weight; WT120: weight at 120 days of age; WT180: weight at 180 days of age; WT270: weight at 270 days of age; WT365: weight at 365 days of age.

## Data Availability

The data sets used in this study are the property of Small Ruminant Training and Research Centre, Pattoki, University of Veterinary and Animal Sciences, Lahore. Data could be requested with permission from the concerned institute.
